# A Technique for the Internalization of Nephroureteral Stents in Patients With Ileal Conduits

**DOI:** 10.7759/cureus.75131

**Published:** 2024-12-05

**Authors:** Marliza O'Dwyer, Kevin Sheahan, Caroline E Kelly, Niall McEniff, John Mark Ryan

**Affiliations:** 1 Department of Interventional Radiology, St James's Hospital, Dublin, IRL; 2 Department of Interventional Radiology, St James’s Hospital, Dublin, IRL; 3 Department of Urology, Beaumont Hospital, Dublin, IRL

**Keywords:** ileal conduit, internalisation, interventional radiology, nephro-ureteric stents, pelvic radiation, urology

## Abstract

We present a method of internalization of nephroureteral stents to internalized ureteral stents in a patient with an ileac conduit urostomy with radiation-induced ureteral strictures, and recurrent urinary tract infections (UTIs). This technique is applicable to patients requiring internalization of nephroureteral stents in the setting of an ileal conduit, emphasizing patient consent, preparation, position, imaging guidance, and antibiotic prophylaxis.

The successful application of this technique offers a practical solution for managing recurrent UTIs in patients with similar medical histories, providing both clinical and procedural insights.

## Introduction

Interventional radiology (IR) plays a pivotal role in the management of complex urological conditions, particularly in cases where traditional approaches prove challenging [1]. This technical note introduces an IR technique that addresses recurrent urinary tract infections (UTIs) and discomfort experienced by patients with nephroureteral stents with an ileal conduit urostomy. This technique demonstrates a practical solution to internalizing nephroureteral stents to ureteral stents in a patient with an ileal conduit. Our patient also highlights the clinical utility of this technique to avoid complications with external nephroureteral stents.

## Case presentation

**Figure 1:**
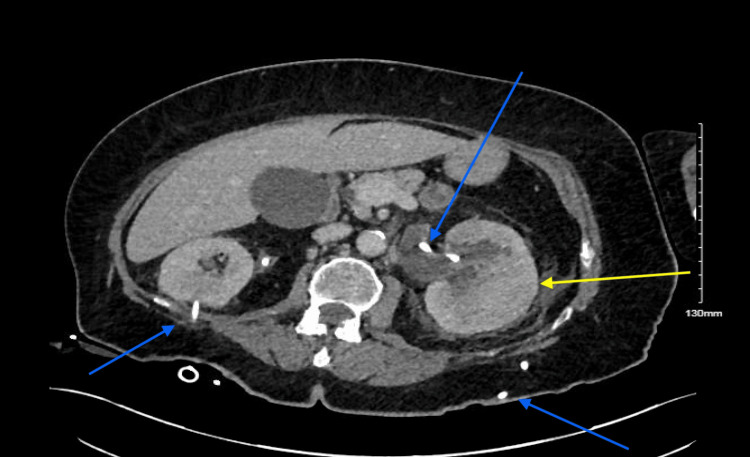
CT urogram performed three months before procedure demonstrating left-sided pyelonephritis (yellow arrow) and bilateral nephroureteral stents (blue arrows) in situ.

A 61-year-old female patient was presented with a challenging medical history of a hysterectomy, lymph node dissection, and adjuvant radiotherapy in 2009 for curative treatment of stage IB squamous cell carcinoma of the cervix. Unfortunately, this resulted in a right ureteral stricture. Complications further ensued with the need for nephrostomy placement, ureteral stent insertion, and an ileal conduit urinary diversion in August 2018. The patient had long-term bilateral nephroureteral stents, which over prolonged time, were associated with multiple urinary tract infections (Figure 1). The patient also developed skin irritation from urinary leaks at the skin entrance site and significant discomfort from the nephroureteral stents.

We hypothesized that if the nephroureteral stents were long enough, they could easily be changed retrogradely every three months, allowing the patient to live without the external nephrostomy component of the stents. An alternative approach used at our institution involves retrogradely cannulating the ureters from the ileal conduit and placing pigtail stents, similar to the study by Maher et al. [1]. However, retrograde placement without endoscopic guidance is technically more challenging and time-consuming than antegrade stent placement, particularly in the setting of distal ureteric strictures.

The patient's current status included bilateral flank pain and skin excoriation, worse on the left side, necessitating bilateral nephroureteral stent exchange and internalization of the ureteral stents. Prior imaging was reviewed.

Patient consent

Comprehensive discussions and informed consent were obtained from the patient. Risks included losing access requiring nephrostomy reinsertion, displacement of the ureteral stents, and hematuria [2,3]. Written informed consent was also obtained from the patient for publication of this technique.

Patient preparation and procedure

**Figure 2:**
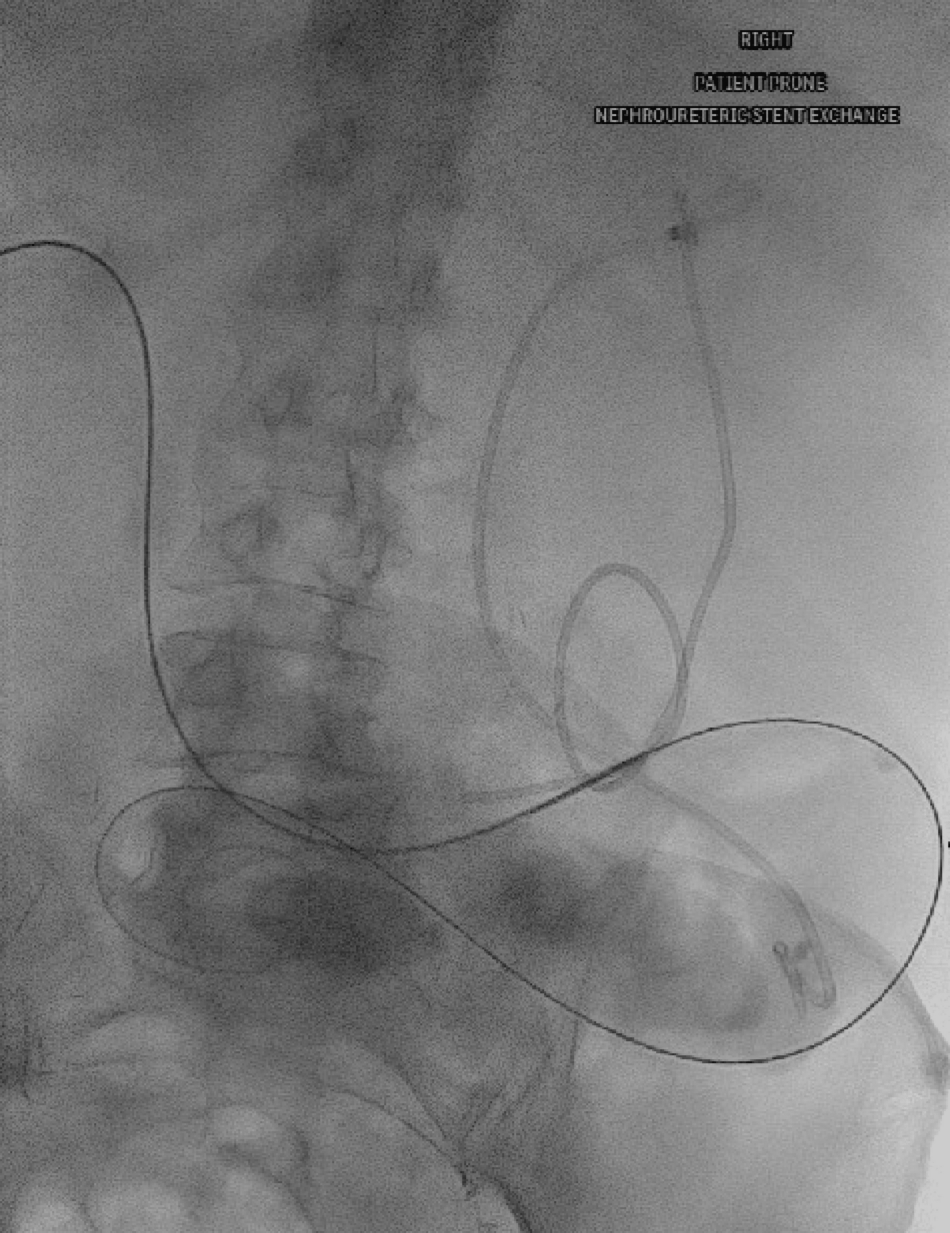
With the patient prone a wire was inserted into the left nephroureteral stent and into the ileal conduit.

Antibiotic prophylaxis was administered and pre-procedure imaging, blood tests, and medications were reviewed. The patient was positioned prone on the fluoroscopic imaging table in the interventional radiology suite. The bilateral nephroureterostomies were prepped and draped with Betadine. Periprocedural sedation was given by the nursing staff using fentanyl and midazolam. The right nephroureterostomy was approached first, followed by the left. Injection of contrast into the stent under fluoroscopic guidance was performed. The position of the nephroureteral stent was confirmed. A 0.35", 180 cm stiff Terumo wire (Somerset, NJ: Terumo Medical) was inserted into the nephroureteral stent and into the ileal conduit (Figure 2).

**Figure 3:**
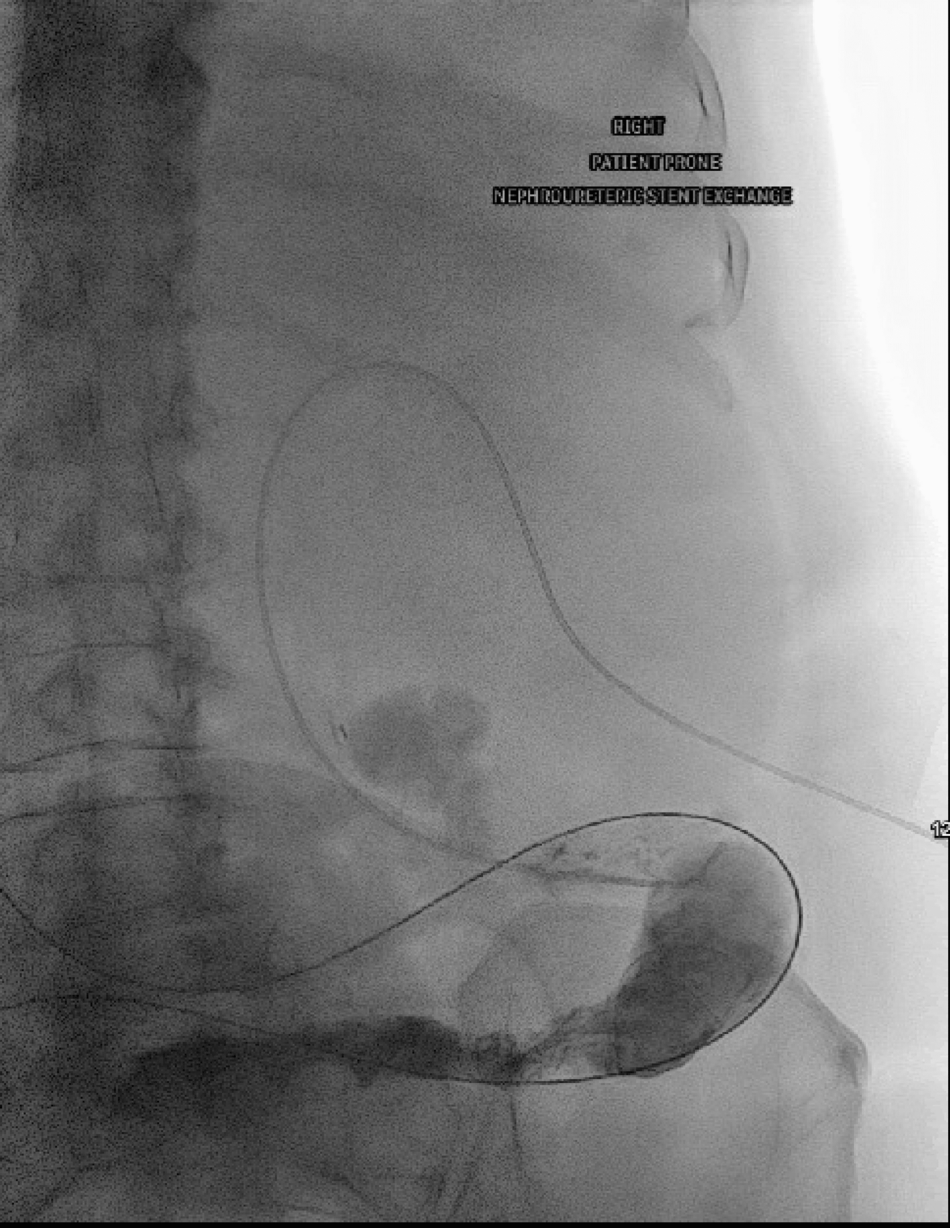
Contrast injection via the Berenstein catheter outlined the ileal conduit. Berenstein catheter (Bloomington, IN: Cook Medical)

**Figure 4:**
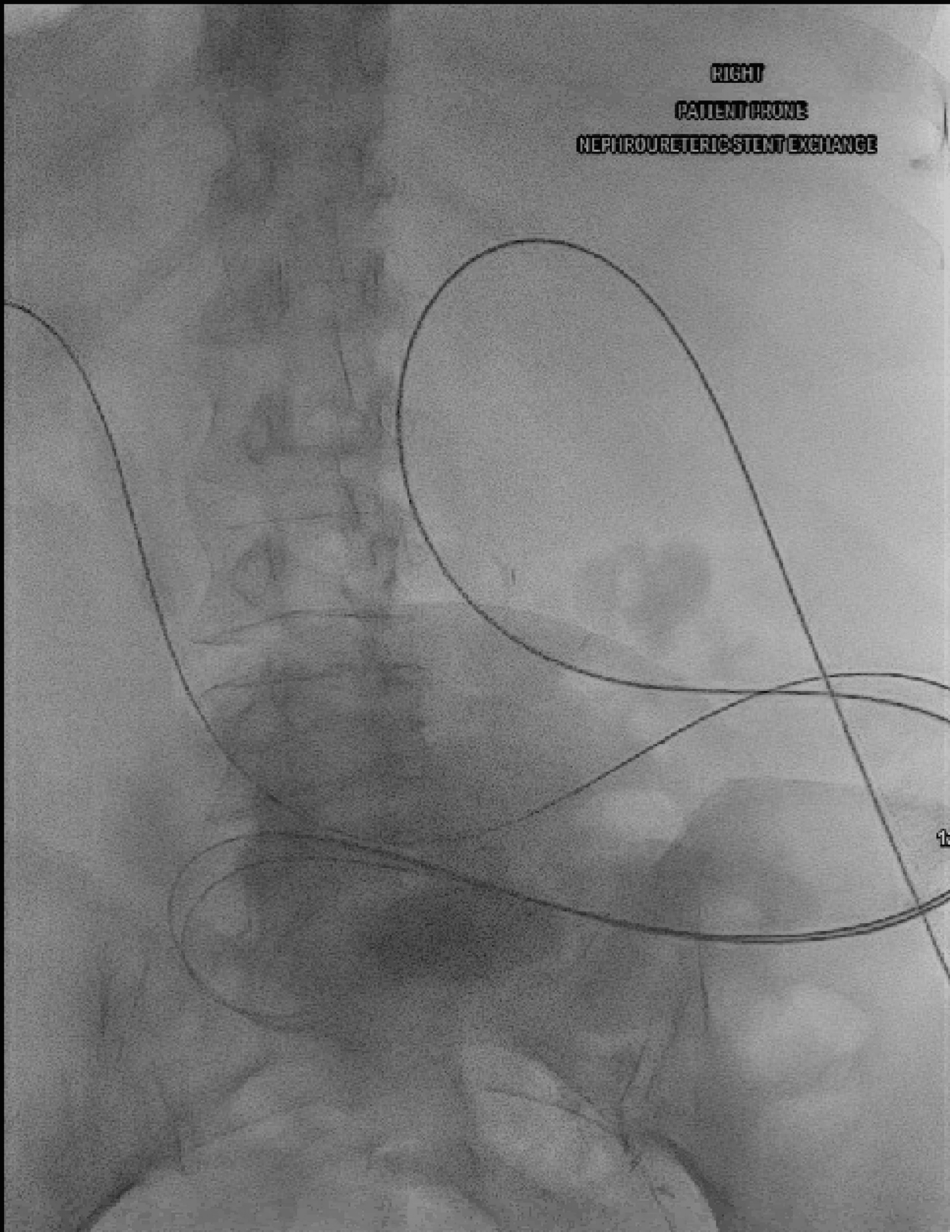
Amplatz wire was placed through the catheter and into the ileal conduit stoma bag. Amplatz wire (Marlborough, MA: Boston Scientific)

The nephroureteral stent was removed over the wire and exchanged for a 65 cm Berenstein catheter (Bloomington, IN: Cook Medical). The Terumo wire was removed and contrast injection via the Berenstein catheter outlined the ileal conduit (Figure 3). A 0.35" super stiff Amplatz wire (Marlborough, MA: Boston Scientific) was placed through the catheter and into the ileal conduit stoma bag (Figure 4). 

**Figure 5:**
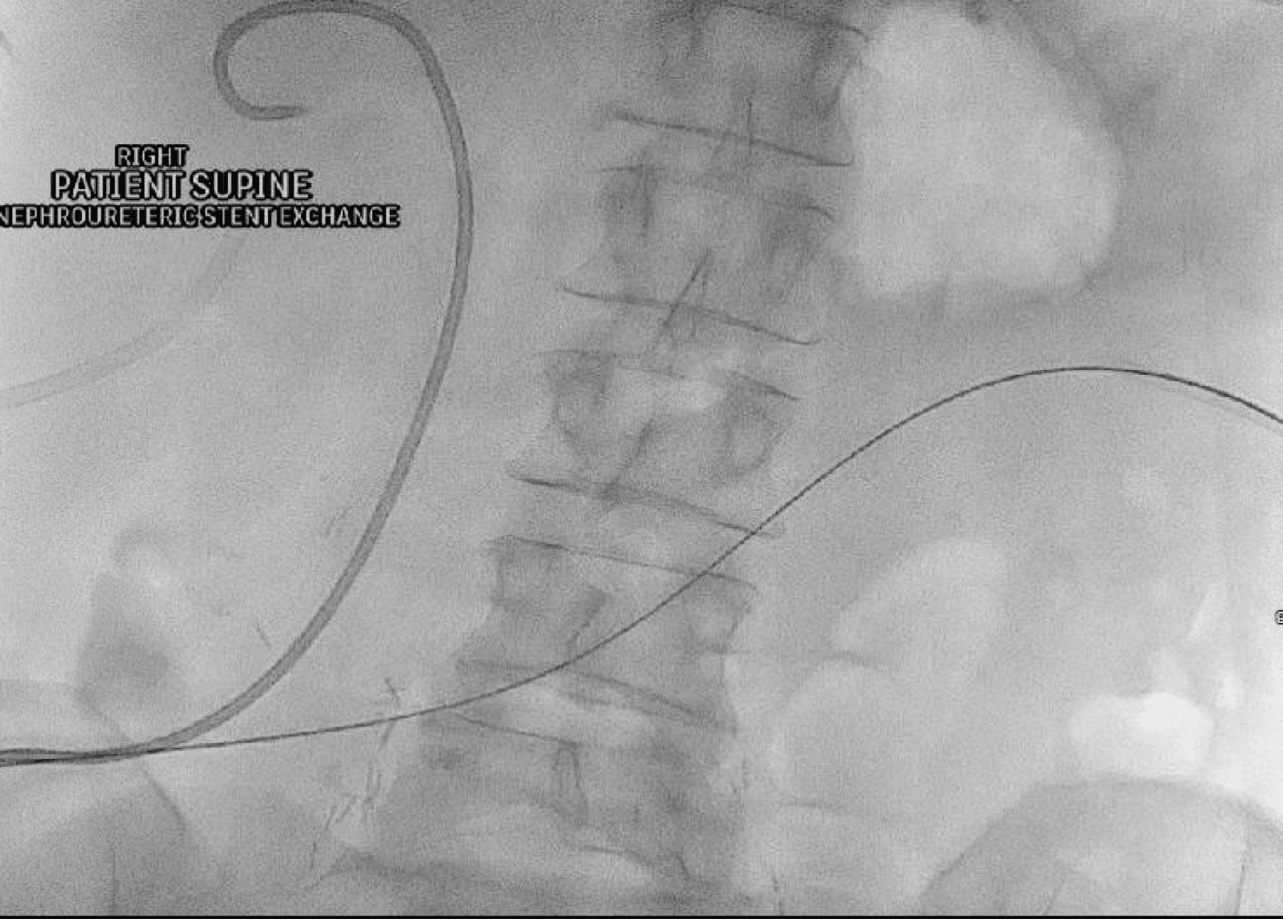
New 8 Fr right ureteral stent in satisfactory position after being placed over the Amplatz wire with proximal pigtail in the renal collecting system. Amplatz wire (Marlborough, MA: Boston Scientific)

An 8 Fr angiographic percutaneous sheath was placed into the renal pelvis to secure the wire access to the kidney. Bilateral Amplatz wires were secured for change of position with tape. The patient turned into the supine position and the ileal conduit stoma bag was removed and prepped with povidone-iodine (Betadine). Lidocaine hydrochloride gel (Instillagel; Melsungen, Germany: CliniMed) was administered to the stoma. The super-stiff Amplatz wires were secured at the ileal conduit site with a 10 Fr angiographic sheath. New 8 Fr × 26 cm regular double-J ureteral stents (Boston Scientific) were sequentially placed over the Amplatz wires under fluoroscopic guidance and the pigtails formed in each renal pelvis (Figure 5).

**Figure 6:**
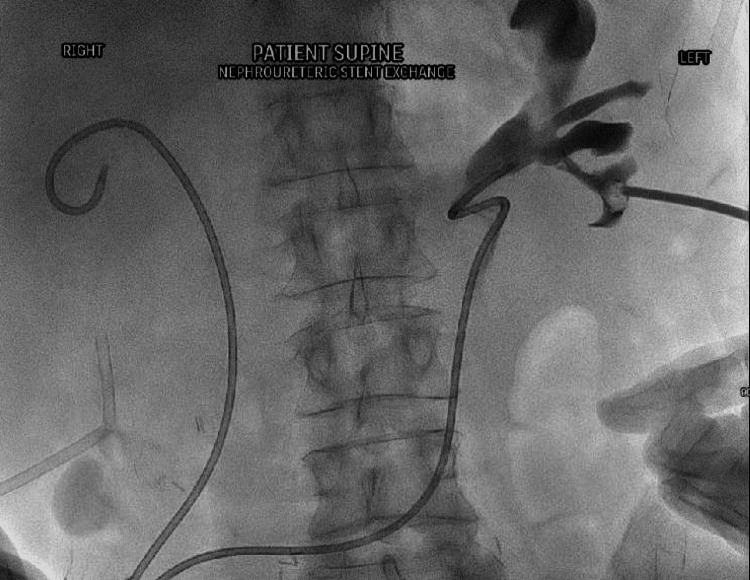
The left angiographic sheath injected with contrast to confirm position of the proximal left ureteral stent.

**Figure 7:**
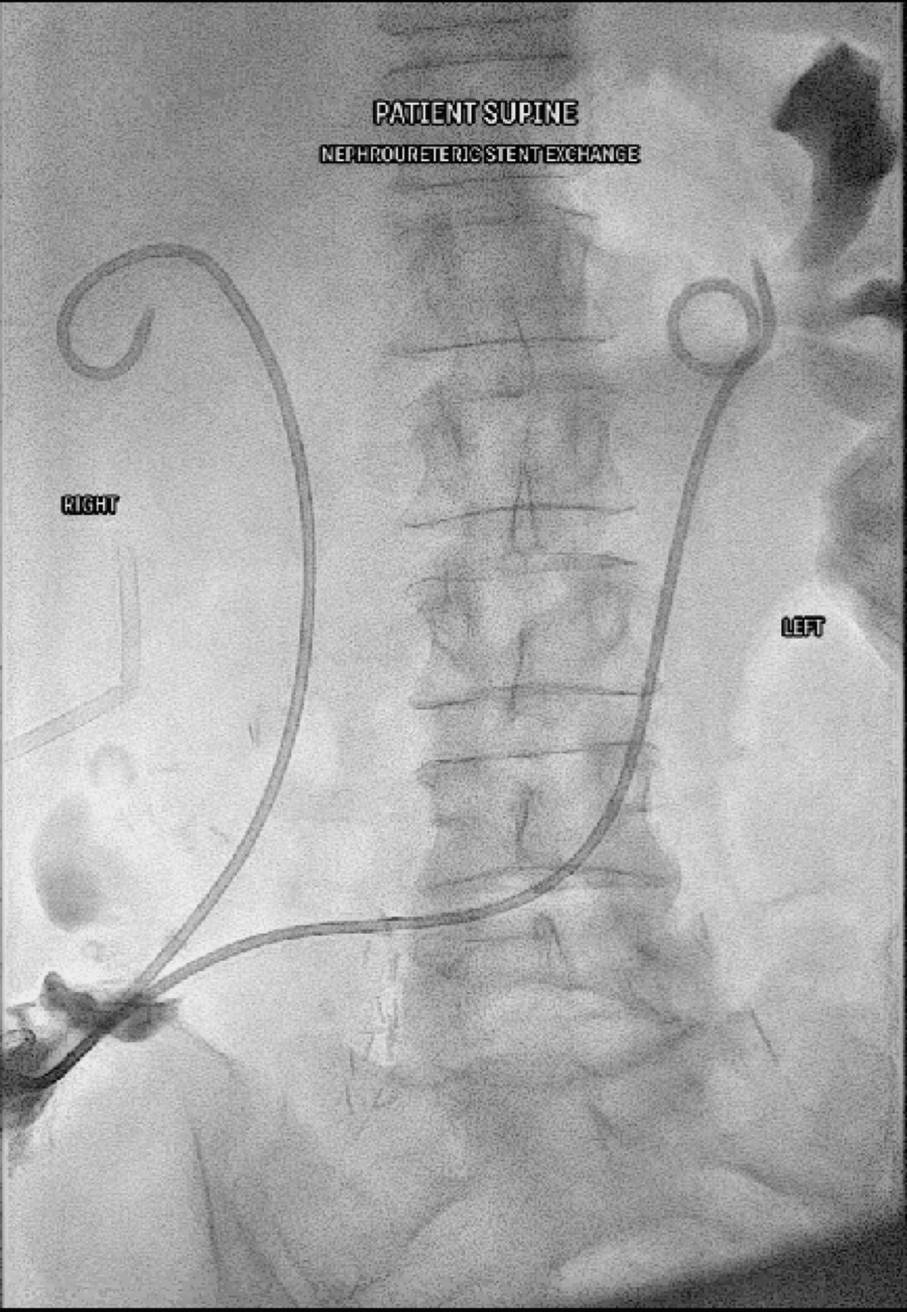
Final fluoroscopic image confirming position of internalized ureteral stents.

Contrast injection via the angiographic sheath within the renal pelvis was used to confirm the position of the proximal left ureteral stent (Figure 6). The wire was removed and ureteral stents were confirmed to be in a satisfactory position. The proximal and distal sheaths were then removed (Figure 7). The distal pigtails were formed external to the ileal conduit.

The patient tolerated the procedure well. The procedure resulted in the successful internalization of nephroureteral stents to internalized ureteral stents with the distal portions of the stent within the ileal conduit.

## Discussion

This technique of internalizing nephroureteral stents to internalized ureteral stents within an ileal conduit represents a helpful advancement in the management of complex urological cases [1]. The successful application and safety of this technique depend on the patient (e.g., type of ileal conduit, anatomy, and BMI) and technical, environmental, and device factors [4].

Patient factors

Patient-related variables are instrumental in determining the appropriateness and success of the internalization technique. Patient selection remains critical, encompassing overall health, comorbidities, and urinary tract history. Patient willingness to actively participate in post-procedure care is essential for successful outcomes. Adequate patient counseling is vital to establish realistic expectations and ensure active patient engagement.

Technical factors

Technical proficiency plays a paramount role in the execution of the internalization procedure. Interventional radiologists must possess the requisite skills and experience to navigate the intricate steps involved, which include wire insertion, stent removal, and precise stent placement. A commitment to maintaining meticulous sterile technique and fostering effective communication among the procedural team, particularly when switching from prone to supine, is equally indispensable.

Device factors

**Figure 8:**
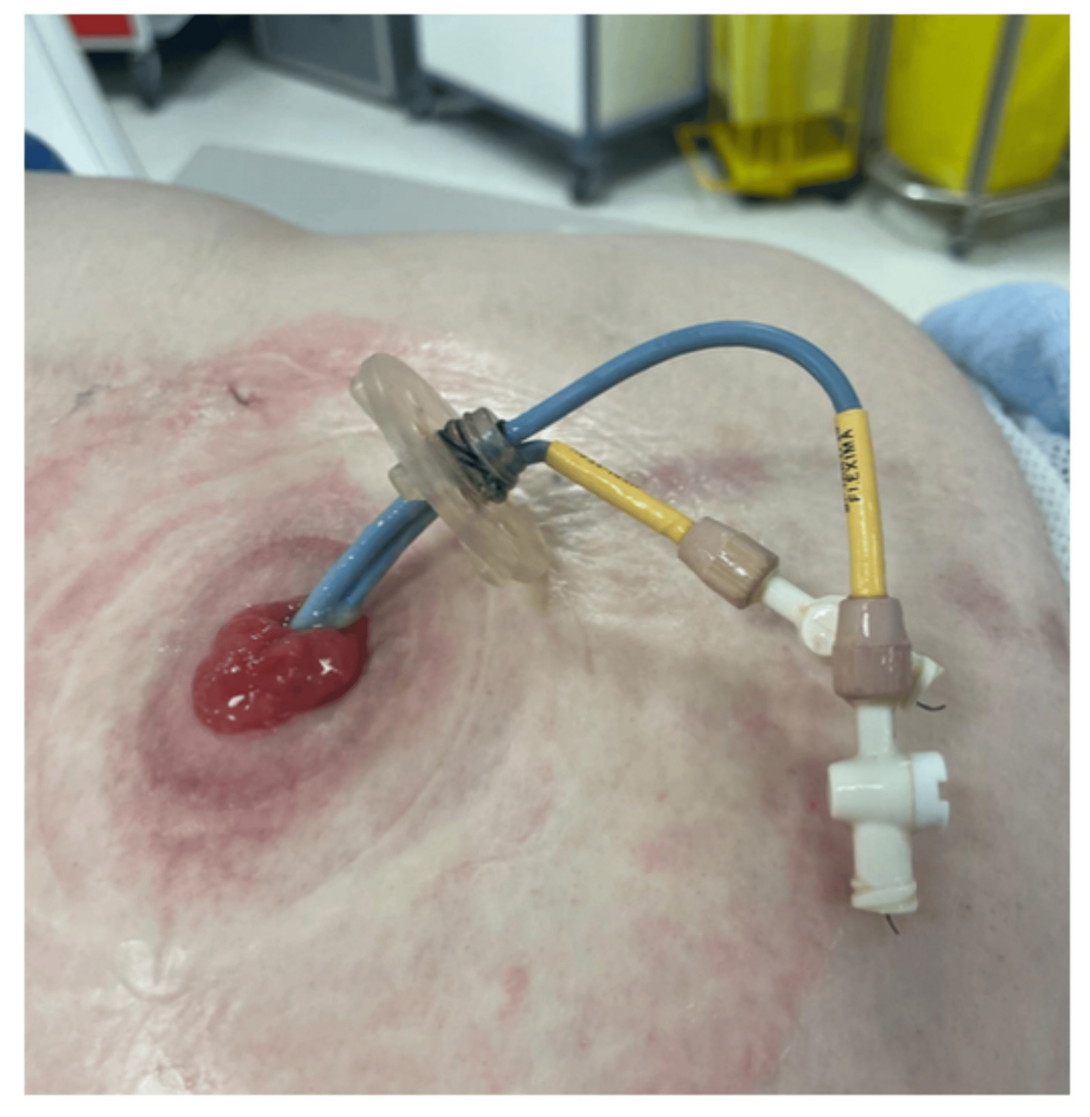
Biliary stents secured with gastrostomy disc in our institution.

The choice of devices and equipment employed during the procedure significantly influences its outcome. Employing high-quality wires, catheters, and stents is crucial for facilitating smooth wire passage, stent exchange, and secure placement. Ongoing advancements in device technology, with a focus on tailoring equipment to the internalization technique, can enhance procedural efficiency and safety [5]. Bander stents are an alternative specialized ureteral diversion stent used in ureteroileal conduit construction which can be 6 or 7 Fr and are polytetrafluoroethylene (PTFE)-covered stents [6-8]. Similar to other devices, these facilitate drainage from the kidney to an external stoma, and they include color-coded radiopaque markers to assist in accurate positioning. The design ensures the stents are secure and can be easily replaced. Another option is to use biliary stents depicted in Figure 8, which are held in place by a gastrostomy disc. Close collaboration between interventional radiologists and device manufacturers holds promise for driving innovations that can further enhance patient outcomes and reduce procedural complexity [5].

Types of ileal conduit

The type of ileal conduit employed also influences the procedure's application and success. The standard ileal conduit (Bricker procedure) is the most common, but variations include the Studer orthotopic neobladder and cutaneous ureteroileostomy [9]. Patients with different conduit types may require tailored approaches for effective stent management. While the internalization technique may not be directly applicable in some cases, the consideration of how to manage stents within these various conduit types remains a crucial aspect of patient care.

Anatomical factors

Anatomical or physiological complexities pose challenges to the procedure's execution. Factors such as ureteral strictures, anatomical variations, peri-conduit adhesions, active infection, inflammation, and obesity can complicate wire passage, stent placement, and overall procedural success [10]. A meticulous pre-operative assessment and procedural planning are essential for addressing challenging anatomical factors, minimizing risks, and ensuring patient safety.

Environmental factors

Environmental factors, including the availability and expertise of support staff, sterility of the operating environment, and accessibility of necessary equipment, play a critical role in the procedure's success.

## Conclusions

In conclusion, the technique of internalizing nephroureteral stents to ureteral stents within an ileal conduit is useful in the management of complex urological cases. Success depends on a nuanced understanding of patient, technical, environmental, and device factors. Additionally, an awareness of the types of ileal conduit and anticipation of challenging anatomical factors is crucial in tailoring the procedure to individual patient needs. As interventional radiology continues to evolve, ongoing collaboration between clinicians and device manufacturers holds promise for further enhancing the technique's safety and efficacy. This technique holds promise for similar cases and highlights the importance of patient-centered approaches in interventional radiology.
